# Autoimmune Atrophic Gastritis: A Clinical Review

**DOI:** 10.3390/cancers16071310

**Published:** 2024-03-28

**Authors:** Chiara Castellana, Leonardo Henry Eusebi, Elton Dajti, Veronica Iascone, Amanda Vestito, Pietro Fusaroli, Lorenzo Fuccio, Antonietta D’Errico, Rocco Maurizio Zagari

**Affiliations:** 1 Department of Medical Sciences and Surgery, University of Bologna, 40138 Bologna, Italy; chiara.castellana@studio.unibo.it (C.C.); leonardo.eusebi@unibo.it (L.H.E.); elton.dajti2@unibo.it (E.D.); veronica.iascone2@unibo.it (V.I.); lorenzo.fuccio3@unibo.it (L.F.); antonietta.derrico@unibo.it (A.D.); 2 Gastroenterology Unit, IRCCS—Azienda Ospedaliero-Universitaria di Bologna, 40138 Bologna, Italy; amanda.vestito@aosp.bo.it; 3 Gastro-Esophageal Organic Diseases Unit, IRCCS—Azienda Ospedaliero-Universitaria di Bologna, 40138 Bologna, Italy; 4 Gastroenterology Unit, Hospital of Imola, 40026 Imola, Italy; 5 Pathology Unit, IRCCS—Azienda Ospedaliero-Universitaria di Bologna, 40138 Bologna, Italy

**Keywords:** autoimmune atrophic gastritis, anti-parietal cell antibodies, gastric carcinoids

## Abstract

**Simple Summary:**

Autoimmune atrophic gastritis can lead to serious conditions, including malabsorption and vitamin deficiencies, that may cause anemia, neurological disorders, and gastric malignancies. This paper provides recent evidence on the pathogenesis, diagnosis, clinical presentation, risk of malignancies, endoscopic surveillance, and treatment of autoimmune atrophic gastritis. This review provides a valuable update for healthcare professionals and researchers, and the findings may help improve the diagnosis and management of patients with autoimmune atrophic gastritis, leading to improved outcomes and shaping future research directions.

**Abstract:**

Autoimmune atrophic gastritis (AAG) is a chronic condition characterized by the presence of atrophy in the oxyntic mucosa due to anti-parietal cell antibodies. This review provides a comprehensive and up-to-date overview of autoimmune atrophic gastritis, reporting recent evidence on epidemiology, pathogenesis, diagnosis, clinical presentation, risk of malignancies, and management. The prevalence of AAG has been estimated at between 0.3% and 2.7% in the general population. The diagnosis of AAG is based on a combination of the serologic profile and the histological examination of gastric biopsies. Patients with AAG are often asymptomatic but can also have dyspeptic or reflux symptoms. The atrophy of the oxyntic mucosa leads to iron and vitamin B12 malabsorption, which may result in anemia and neurological affections. Autoimmune atrophic gastritis is associated with an increased risk of type I neuroendocrine tumors (NETs) and gastric cancer, with an incidence rate of 2.8% and 0.5% per person/year, respectively. Management is directed to reinstate vitamins and iron and to prevent malignancies with endoscopic surveillance. In conclusion, atrophic autoimmune gastritis is an infrequent condition, often asymptomatic and misdiagnosed, that requires an early diagnosis for appropriate vitamin supplementation and endoscopic follow-up for the early diagnosis of NETs and gastric cancer.

## 1. Introduction

Autoimmune atrophic gastritis (AAG) is a chronic condition in which the immune system attacks and damages the oxyntic mucosa of the stomach through the production of anti-parietal cell antibodies and/or anti-intrinsic factor antibodies. Although the pathogenesis of AAG is not entirely defined, there is evidence of a primary involvement of cellular immunity promoted by the CD4+Th1 response; autoantibodies against gastric parietal cells may be the consequence of damage to the H+/K+ ATPase induced by autoreactive Th1 cells [1,2]. The inflammatory response leads to the destruction or loss of parietal cells normally involved in the production of gastric acid and intrinsic factors. Thus, autoimmune gastritis tends to spare the antral mucosa and to affect only the gastric body and the fundus [1,3]. This is in contrast with chronic gastritis related to *Helicobacter pylori* (*H. pylori*) infection, which mainly involves the antral mucosa and, in a subgroup of subjects, also the corpus mucosa, leading to pangastritis. However, the relationship between *H. pylori* infection and AAG is still unclear. It is still debated whether *H. pylori*-related pangastritis and AAG are two clearly separated entities [4].

Autoimmune gastritis is a growing concern in the medical field as it can lead to a range of serious conditions, including malabsorption and vitamin deficiencies, and an increased risk of gastric malignancies, such as carcinoids and cancer.

This study provides a comprehensive and up-to-date review of AAG, reporting recent evidence on epidemiology, pathogenesis, diagnosis, clinical presentation, risk of carcinoids and gastric cancer, and management of this condition.

## 2. Epidemiology

The prevalence of AAG in the general population is still unclear because of the lack of standardized criteria for the diagnosis of this condition. Some studies correctly used histological examination of gastric biopsy specimens for the diagnosis of AAG, whereas in other studies the diagnosis was based only on low levels of Vitamin B12 or serological markers, such as anti-parietal cell antibodies and/or anti-intrinsic factor antibodies.

Autoimmune gastritis is considered a relatively rare condition compared to other gastrointestinal disorders, with a prevalence estimated between 0.3% and 2.7% in the general population [5]. Variations in the prevalence of autoimmune gastritis have been reported among different ethnic groups, geographic regions, and genders. Autoimmune gastritis seems to be more frequent in Western than Eastern countries [1]; however, it cannot be excluded that this finding may be partially due to the higher prevalence of *H. pylori*-related atrophic gastritis, which may lead to a misdiagnosis of AAG in Asian countries. A Japanese study including 10.822 patients found that endoscopists frequently overlooked AAG, especially in patients who underwent an *H. pylori* eradication treatment, thus suggesting that the prevalence of AAG could be higher than that reported [6].

Autoimmune gastritis is associated with female gender, older age, and autoimmune disorders. Autoimmune gastritis is more common in women, with a female:male ratio of 2–3:1, and in subjects aged >60 years [7,8]. The prevalence of AAG is also higher in people with a personal or family history of autoimmune disorders, especially type 1 diabetes and autoimmune thyroiditis, making it five times more frequent in patients with such conditions compared to controls [9]. In particular, AAG seems to be the most frequent autoimmune disease in patients with autoimmune thyroiditis, with a prevalence of 2.8% [10]. On the other hand, a recent retrospective cohort study showed that 36% of AAG patients had autoimmune thyroiditis, followed by rheumatoid arthritis (9%), systemic lupus erythematosus (6%), and celiac disease (3%) [11,12].

## 3. Etiopathogenesis

**Figure 1:**
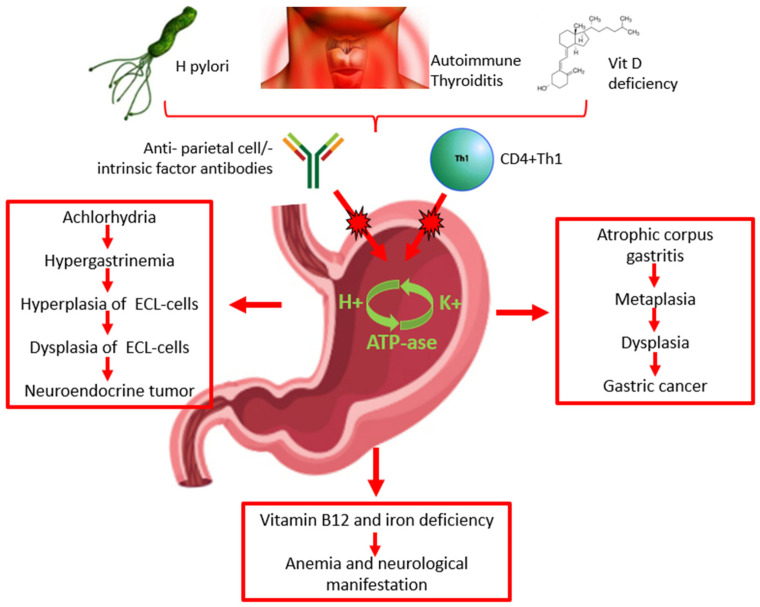
Pathogenesis, diagnosis, clinical presentation of autoimmune atrophic gastritis, and risk of malignancy. ECL: enterochromaffin-like.

The pathogenesis of AAG is still controversial. The characteristic chronic inflammation of the gastric mucosa may be attributable to the presence of anti-parietal cell antibodies, specifically their H+/K+ ATPase and/or anti-intrinsic factor antibodies that are present in the serum of 60–90% and 50–70% of patients, respectively (Figure 1) [13,14]. However, some studies suggest that the anti-parietal cell antibodies may not be directly involved in the pathogenesis of AAG; autoantibodies against gastric parietal cells may be the consequence of damage to the H+/K+ ATPase induced by autoreactive T cells that results in the exposure of molecular patterns leading to the production of anti-parietal cell antibodies (Figure 1) [1,2]. Recent studies have suggested that vitamin D deficiency may be involved in the pathogenesis of autoimmune diseases, including AAG. The vitamin D receptor is involved in the activation and differentiation of T-cells, which is essential for the maintenance of protective immunity and tolerance to self-antigens. Thus, vitamin D deficiency may have consequences on T-cell maturation and function and, consequently, on the risk of the development and progression of autoimmune diseases, including AAG (Figure 1) [15].

The relationship between *H. pylori* infection and AAG is still unclear. *H. pylori* may play a role in the induction and/or exacerbation of AAG (Figure 1). *H*. *pylori*-positive patients with corpus atrophic gastritis seem to have high levels of anti-parietal cell antibodies, which decrease significantly after *H. pylori* eradication. The destruction of parietal cells by *H. pylori*-related inflammation may lead to the exposure of molecular patterns of the ATPase pump H+/K+ and consequently the production of anti-parietal cell antibodies as a consequence of a cross reaction between molecular antigens of the bacterium and molecular patterns of the ATPase pump H+/K+; this is more likely to occur in genetically predisposed subjects with specific MHC class II haplotypes [16] (Table 1). Two recent case reports showed that *H. pylori*-related gastritis ultimately transitioned into AAG; furthermore, they showed that the progression of AAG caused a spontaneous disappearance of *H. pylori* infection as a consequence of the worsening of the gastric atrophy [17].

On the other hand, a study on a murine model supported the hypothesis that *H. pylori* infection may suppress the development of AAG. It was demonstrated that AAG is promoted by a CD4+ Th1 response that seems to be downregulated in mice infected by *H. pylori* due to Th2-type immune responses and transforming growth factor β [18]. This would be in line with a recent case report showing that a 73-year-old woman affected by *H. pylori*-related gastritis developed AAG after eradication therapy with rapid progression of the atrophic pattern in the corpus within three years. The authors concluded that *H. pylori* gastritis may have suppressed AAG activity until eradication occurred [19]. Finally, the prevalence of AAG is very low in Asian countries, where there is a high prevalence of *H. pylori* infection [18].

Autoimmune gastritis results in a combination of genetic and environmental factors. As previously mentioned, a significant predisposition is present for individuals with a family history of autoimmune disorders. Indeed, the association between AAG and other autoimmune disorders supports the importance of genetic susceptibility, such as the presence of specific HLA haplotypes [10]. The pathogenetic relationship between autoimmune thyroiditis and autoimmune gastritis remains to be clarified. The most supported hypothesis suggests the existence of an immunological cross-reaction. Indeed, a common molecular pattern between thyroid peroxidase and H+/K+ ATPase in gastric parietal cells has been described (Figure 1) [27].

Finally, Winter et al. reported that mice with chemokine receptor type 7 (CCR7) deficiency developed autoimmune gastritis [28]. The receptor CCR7 is a protein of the G-protein-receptor family able to activate B and T lymphocytes and stimulate the maturation of the dendritic cells, which are essential for the maintenance of protective immunity and tolerance to self-antigens [29].

## 4. Diagnosis

The diagnosis of AAG is based on the combination of the serologic profile and the histological examination of gastric biopsies (Figure 1).

Serologic markers of AAG include anti-parietal cell antibodies and anti-intrinsic factor antibodies [30]. The sensitivity and specificity of anti-parietal cell antibodies for the diagnosis of AAG are 81% and 90%, respectively, whereas anti-intrinsic factor antibodies have poor sensitivity (27%) and high specificity (100%) [1]. Using a prevalence of AAG of 3% in the general population [5], the negative predictive value of anti-parietal cell antibodies for the presence of AAG is very high, being 99%, while the positive predictive value is very low, only 20%. Thus, serum anti-parietal cell antibodies are a very powerful test to rule out AAG; only a few subjects with a negative test will suffer a missed diagnosis of AAG with a consequent delay in treatment. Anti-parietal cell antibodies may appear a long time before the development of AAG. Several subjects with positive anti-parietal cell antibodies do not have AAG on histology. Similarly to coeliac disease, a new concept of “potential” AAG has recently emerged; potential AAG was defined as the presence of anti-parietal cell antibodies in the absence of gastric histopathological atrophy (at any site) and the absence of current *H. pylori* infection. It has been reported that about 50% of subjects with “potential” AAG will develop an overt AAG over a median time of two years [31] (Table 1). A recent prospective study including 93 patients evaluated the natural history of “potential” AAG during a median follow-up period of 52 months. The study reported an annual rate of progression of 10.9% (95% CI 7.8–15.2) from “potential” AAG to “overt” AAG, confirming the hypothesis that anti-parietal cell antibodies are a true marker of “potential” AAG in patients without corpus atrophy [20] (Table 1). However, early histopathological markers of “potential” AAG have not yet been described. A recent study described increased CD3+ intraepithelial lymphocyte infiltration compared to healthy controls and *H. pylori* gastritis in the face of architecturally normal mucosa [32]. Potential predictors of progression (i.e., gender, age, association with other autoimmune diseases) to overt AAG are still debated, and larger-scale studies are needed. However, serum anti-parietal cell antibodies decline with age and thus may be less reliable in elderly patients [5].

Serum pepsinogens I and II are useful in the diagnosis of AAG. Pepsinogen I (PG-I) is produced by the oxyntic mucosa, while pepsinogen II (PG-II) is produced by both the oxyntic and antral mucosa. Thus, low serum levels of PG-I (<70 μ/L) or a low ratio of PG-I/PG-II (<3) indicate the presence of corpus atrophic gastritis with a sensitivity and specificity of 69% and 88%, respectively [33]. On the contrary, serum gastrin, which is synthesized by antral G cells, is often high in patients with autoimmune gastritis as a consequence of the hypo-achlorhydria due to the atrophy of the oxyntic mucosa [34]. Combining these serum tests led to the development of another useful serological tool for the diagnosis of corpus atrophic gastritis based on the serum levels of pepsinogens, gastrin-17, and anti-*Helicobacter pylori* antibodies (Gastropanel^®,^, Biohit Oyj, Helsinki, Finland). Low levels of serum PG-I or ratio PG-I/PG-II and high levels of gastrin-17 (G-17) indicate the presence of corpus atrophic gastritis. Pooling data from the seven studies that assessed the performance of the panel test for the diagnosis of corpus-limited atrophic gastritis, the summary sensitivity and specificity were 70.4% and 98.4%, respectively [35].

In patients with AAG, serum chromogranin A is also abnormally high. However, chromogranin A can be elevated in multiple conditions, and it is not recommended as a diagnostic test for autoimmune gastritis [36].

An accurate endoscopic examination is mandatory in patients with suspected AAG. Endoscopic findings in AAG include mucosal erythema, nodularity, and an ipo-atrophic mucosal pattern. However, white-light endoscopy has some limitations for detecting mucosal atrophy. New advanced endoscopic techniques, such as high-definition (HD) endoscopy, chromoendoscopy (dye or virtual based, including narrow-band imaging), magnification, and autofluorescence, allow the detection of minimal changes in the mucosal pattern. In normal conditions, micro-vessels of the gastric mucosa of the body show a honeycomb subepithelial capillary network and a regular-shape venous collection, which are absent in patients with atrophic gastritis [37]. However, endoscopy alone is not sufficient for a diagnosis and should be combined with histological evaluation.

Histological evaluation of gastric biopsies is the gold standard for the diagnosis of AAG [8]. According to recent European guidelines, MAPS II, at least two biopsies from the antrum and two biopsies from the corpus should be taken and collected in separate vials [38]. According to the Updated Sydney System, one more biopsy specimen should be taken from the incisura angularis [39].

**Figure 2:**
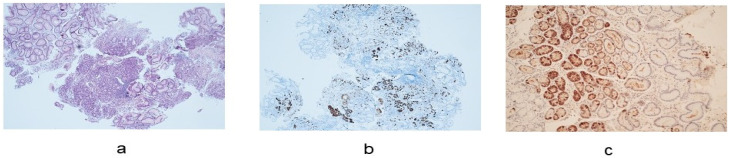
Histological features of autoimmune atrophic gastritis. (**a**). Oxyntic mucosa with diffuse pseudopyloric and intestinal metaplasia (hematoxylin-eosin staining) (10× magnification). (**b**). Intraglandular linear and nodular hyperplasia of the enterochromaffin-like cells (chromogranin A staining) (20× magnification). (**c**). Hyperplasia of gastrin G cells in antrum (hematoxylin-eosin staining) (20× magnification).

The histological diagnosis of AAG is characterized by the presence of atrophy only in the corpus mucosa. Three steps of inflammation of the oxyntic mucosa can also be present. The early phase is characterized by lamina propria inflammation with a prevalence of lymphocyte CD4+ and a large variety of pseudopyloric or pancreatic acinar metaplasia. In this phase, atrophy may be mild or moderate, and residual parietal cells can be hypertrophic due to an excess of gastrin. The florid phase is characterized by moderate or severe atrophy of the oxyntic glands with an inflammatory response mediated by lymphoplasma cells, enterochromaffin-like cell hyperplasia, and predominant intestinal metaplasia in the oxyntic mucosa, whereas antral biopsy specimens show gastrin cell hyperplasia (Figure 2). Finally, the end phase exhibits severe oxyntic gland loss, intestinal metaplasia, enterochromaffin-like cell hyperplasia, and reduced mucosal inflammation. Enterochromaffin-like cell hyperplasia can be linear or nodular, and it is a precursor of type 1 carcinoid tumors [2].

The AAG is often underdiagnosed in patients with precancerous conditions, such as atrophic gastritis and intestinal metaplasia in the antrum and/or corpus. A recent study including 256 subjects with atrophic gastritis and intestinal metaplasia and 70 control subjects showed a prevalence of AAG of 18% in patients with precancerous conditions and 7% in controls, with a statistically significant difference among the two groups (*p* = 0.033); the prevalence of active or previous infection by *H. pylori* between the two groups was similar. The detection of anti-parietal cell antibodies and the use of serum gastrin, PGI/PGII ratio, and vitamin B12 can be useful to identify patients with AAG among those with precancerous conditions (Table 1) [21].

Finally, given the association between autoimmune gastritis and other autoimmune diseases, screening for conditions like thyroid disorders or type 1 diabetes may be recommended in patients with AAG. On the other hand, clinicians should screen patients with autoimmune thyroiditis for the presence of AAG to enhance the number of occult gastric atrophies diagnosed [40].

## 5. Clinical Manifestations

Patients with AAG are often asymptomatic, but they can also have dyspeptic or reflux symptoms (Table 1) [22]. Symptoms can occur intermittently or persistently and may be triggered or worsened by certain foods or when the stomach is empty. A study including 379 patients with AAG found that 57% of subjects had dyspeptic symptoms that were more frequent in young people, women, non-smokers, and those without pernicious anemia [41]. The etiopathogenesis of dyspepsia is unknown; however, hypochlorhydria and hypergastrinemia may delay gastric emptying, causing post-prandial fulness or early satiety [42,43]. A subgroup of patients with AAG refers to heartburn and regurgitation [44]. However, impedance-pH monitoring in subjects with AAG does not usually show abnormal acid exposure, suggesting that reflux symptoms may be due to non-acid refluxes or may have a functional origin [45].

Pernicious or iron deficiency anemia is frequently associated with AAG (Figure 1). Pernicious anemia is due to a deficiency of vitamin B12 (cobalamin), which is able to regulate the mitotic process of erythrocytes [46] and is associated with an increased corpuscular volume of erythrocytes. Malabsorption of vitamin B12 is a direct consequence of oxyntic mucosal atrophy, since gastric parietal cells are responsible for the production of the intrinsic factor, which is crucial to the absorption of vitamin B12 in the terminal ileum [47,48,49]. Iron deficiency anemia is another hematologic condition typically found in AAG patients and may precede the development of pernicious anemia. It is related to the hypochloridria present in patients with AAG, which is associated with failure to reduce the iron from Fe^3+^ to its absorbable form Fe^2+^ (Table 1) [23].

Moreover, vitamin B12 deficiency may cause fatigue, weakness, and neurological disturbances. Indeed, cobalamin acts as a coenzyme in the reactions involved in the metabolism of fatty acids, and a blockage of these reactions can cause nerve demyelination with consequent axonal damage. As a consequence, peripheral neuropathies can develop, ranging from paresthesia to paraparesis (Figure 1) [50].

A large case series of 168 females showed that AAG can be linked to infertility, recurrent miscarriage, congenital abnormalities, and several obstetric complications. Deficiency of vitamin B12 is involved in pregnancy outcomes, but this is the first study investigating the association between AAG and pregnancy complications [24]. Autoimmune atrophic gastritis is relevant even for repeated pregnancy loss when framed in polyautoimmunity, in particular if antiphospholipid syndrome has been excluded [51]. Thus, AAG may be a relevant comorbidity for women seeking pregnancy and should be ruled out in these subjects (Table 1) [24].

## 6. Risk of Malignancy

Autoimmune atrophic gastritis is a preneoplastic condition that can potentially lead to type I neuroendocrine tumors (NET) and gastric adenocarcinoma [52] (Figure 1).

**Figure 3:**
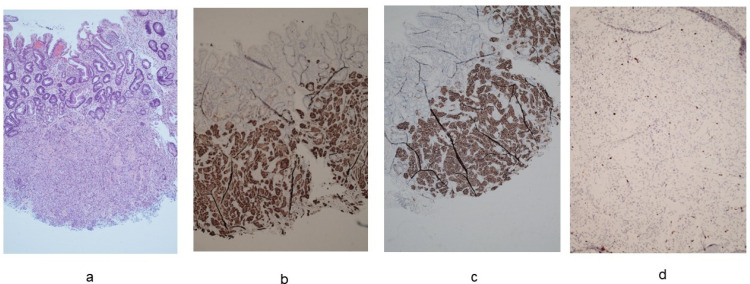
Neuroendocrine tumor (NET) G1, diameter: 3 mm, ki-67: 1.4%. (**a**). Hematoxylin-eosin staining (20× magnification). (**b**). Immunohistochemical staining for synaptophysin (20× magnification). (**c**). Immunohistochemical staining for SSTR2 (20× magnification). (**d**). Immunohistochemical staining for ki67 (40× magnification).

Type I NETs, or carcinoids, are neoplasms that arise from the ECL-cells (Figure 3). The loss of parietal cells and consequent achlorhydria leads to hypersecretion of gastrin by antral G cells. Hypergastrinemia stimulates ECL-cells to produce histamine, resulting in a trophic effect on ECL cells [36,53]. First, linear and then nodular hyperplasia of ECL-cells develop, followed by dysplasia that may progress to a neuroendocrine tumor. The finding of glandular polyps in the gastric body of patients with autoimmune gastritis is strongly associated with the presence of type I carcinoid [54]. In patients with AAG, the odds ratio of developing gastric type I NETs is about 11 [52]. Three recent large monocentric cohort studies assessed the incidence of type I NETs in patients with AAG in Italy [20,25,26].

The study by Rugge et al. showed a cumulative incidence of 4.7% at 2 years of follow-up [25], while Miceli et al. reported an incidence rate of 4.8% after about 4 years (Table 1) [20]. The study by Dilaghi et al. reported a crude incidence of 15.3% at a median follow-up of 5 years; the incidence rate was estimated to be 2.8% per person/year [26]. However, type I carcinoids have a good prognosis, with a rate of metastasis <10% in small (<2 cm) NETs [55].

The association between AAG and the risk of gastric adenocarcinoma is still under debate [56]. Patients with AAG seem to have a three times higher risk of developing gastric adenocarcinoma compared to the general population [55,57]. However, most studies investigated gastric cancer risk in patients with pernicious anemia, especially in elderly people; as pernicious anemia is associated with more severe AAG, the results of such studies may suffer from a selection bias.

A cohort study including 275 patients with AAG assessed an incidence rate of gastric cancer/high grade dysplasia of 0.5% per person/year; risk factors for gastric cancer were age >60 years (hazard ratio (HR) = 4.7), presence of intestinal metaplasia with absence of pseudopyloric metaplasia (HR = 4.3), and presence of pernicious anemia (HR = 4.3) [26] (Table 1).

Gastric adenocarcinoma is a consequence of progressive changes in the gastric mucosa that can induce a slow metamorphosis of the epithelium from healthy to neoplastic. The stages of this process, named Correa cascade, can be summarized in five steps: inflammation, metaplasia, atrophy, dysplasia, and carcinoma [58]. Severe atrophy has a higher risk of developing gastric cancer compared with mild atrophy [55]. Furthermore, hypochloridria results in an alteration of the composition of the gastric microbiota [59], and patients with AAG have higher microbial diversity with an abundance of Streptococci [60] that are also found in individuals with gastric cancer; however, further studies are necessary to confirm the relationship between microbiota changes and gastric cancer [23].

On the other hand, the cohort study by Rugge et al. investigated the risk of gastric cancer in *H. pylori*-negative patients with AAG, including a total of 211 subjects prospectively followed up for a mean of 7.5 years (Table 1) [25]. During the study period, no invasive gastric malignancies, excluding NETs, were detected. The authors demonstrated that the metaplasia found in AAG patients is more often represented by pseudopyloric or complete intestinal metaplasia. As incomplete intestinal metaplasia is considered the lesion with the highest risk for progression to gastric adenocarcinoma, the authors speculate that AAG patients, lacking incomplete intestinal metaplasia, are not at risk of developing gastric adenocarcinoma. Thus, the authors concluded that AAG does not increase the risk of gastric cancer; indeed, the risk of gastric cancer reported in patients with AAG could plausibly result from an unrecognized previous/current *H. pylori* infection. Similar results were reported by another prospective cohort study aimed at evaluating the natural history of autoimmune gastritis in 498 patients with AAG at any stage. During the median follow-up time of 52 months, newly onset neoplastic complications occurred in 41/498 patients (8.5%); in particular, NETs [30] and epithelial dysplasia (18), but no cases of gastric adenocarcinoma, were reported [20]. Murine models showed that M2-macrophages are a pro-carcinogenic immune infiltrate that is crucial for the progression from metaplasia to dysplasia [61]. A M2-macrophage infiltrate is usually found in *H. pylori*-related gastritis, while significantly fewer macrophages were found in AAG patients without *H. pylori* infection [62]. Thus, the lack of macrophages may lead to a more benign pattern of intestinal metaplasia in AAG patients, which may partially explain why AAG does not predispose to gastric adenocarcinoma [63].

## 7. Management

Autoimmune atrophic gastritis does not benefit from specific treatment. Due to the side effects of long-term systemic corticosteroid use, steroids cannot be used in clinical practice. Locally acting anti-inflammatory agents that restrict their effect on the oxyntic mucosa are under development, and new treatments should reduce gastric inflammation and prevent the development and progression of atrophy and intestinal metaplasia.

Proton pump inhibitors may not be useful in AAG patients and instead can worsen iron and nutrient malabsorption. Thus, H2 receptor antagonists (i.e., famotidine), which are less potent than proton pump inhibitors, could provide relief of heartburn and epigastric burning without worsening iron malabsorption. Other potential therapeutic options in AAG patients are represented by sucralfate and other mucosal protective agents that work by promoting mucosal healing thanks to the production of a protective barrier on the gastric mucosa [64].

Ghrelin, a hormone primarily produced in the stomach that regulates appetite and gastric motility, is a potential therapy for improving upper gastrointestinal symptoms in autoimmune gastritis [65]. Further research is now being addressed to evaluate the role of fecal transplantation or prebiotic use in restoring gastric microbiota in AAG patients, as the gastric environment in such patients is compromised due to hypo-achlorhydria [66].

The management of patients with AAG aims to correct iron and vitamin deficiencies and provide an early diagnosis of pre-neoplastic and neoplastic lesions with appropriate endoscopic follow-up.

Vitamin B12 deficiency can be successfully treated with intramuscular or sublingual vitamin B12 supplementation. Unfortunately, neurological symptoms regress only if the supplementation is carried out promptly; otherwise, the damage becomes inexorably progressive [67].

The ESGE guidelines (MAPS II) recommend that patients with AAG undergo endoscopic surveillance with antral and corpus biopsies every 3 to 5 years [38]. To ensure high-quality endoscopic assessment, high-definition endoscopy with dye based or virtual chromoendoscopy (i.e., narrow band imaging) should be performed [68], and any visible lesion should be classified according to the Paris classification [69]. However, studies are underway to try to detect gastric cancer at an early stage through non-invasive techniques. In this regard, some non-coding RNA has been identified but is not yet applied in clinical practice [70].

According to the most recent ESGE guidelines, endoscopic resection of type 1 NETs is indicated if they grow larger than 10 mm [71]. Endoscopic ultrasound can be useful to evaluate tumor invasion and local lymph node metastasis in larger tumors. After resection, endoscopic follow-up every 6 to 12 months is suggested [71].

The medical treatment of NETs with a gastrin-receptor antagonist, named netazepide, has been evaluated. Netazepide reduces serum chromogranin A levels and thus could determine the numerical and dimensional reduction of NETs [72,73]. Another option is the use of somatostatin analogues. In a prospective study including 107 patients with AAG, somatostatin analogues reduced serum levels of gastrin and chromogranin A and led to the disappearance of gastric carcinoids after a median length of treatment of 12 months [74]. However, treatment with netazepideor somatostatin analogues should be continuous because the tumors will regrow if they are stopped [72,74].

## 8. Conclusions

The epidemiology of AAG is still unclear, as this condition is often misdiagnosed, especially in subclinical forms, and is potentially underestimated due to the absence of standard diagnostic criteria. The etiopathogenesis of AAG is not well defined, requiring further studies investigating the possible pathogenesis of this condition.

Recently, a new concept of “potential” AAG has been introduced, defined by the presence of anti-parietal cell antibodies in the absence of gastric histopathological atrophy and *H. pylori* infection. More studies are needed to investigate the natural history of AAG and to highlight possible risk factors associated with a more rapid and severe evolution of the disease. While the association between NETs and AAG is well known, the risk of gastric cancer is still under debate. Several cohort studies with a long follow-up on AAG patients reported the absence of gastric cancer cases and explained how the presence of gastric cancer could plausibly result from previous/current *H. pylori* infection rather than AAG.

Autoimmune atrophic gastritis does not benefit from specific treatment, and the management of patients with AAG aims to correct iron and vitamin deficiencies; therefore, an early diagnosis of AAG is required to guarantee appropriate iron and vitamin B12 supplementation, possibly before the onset of anemia and neurological symptoms. An appropriate endoscopic follow-up is required for the early detection of gastric neoplasia in patients with AAG. New treatments able to reduce gastric inflammation and prevent the development and progression of atrophy are needed.

## Figures and Tables

**Table 1:** Recent findings in the topic of atrophic autoimmune gastritis.

References	Finding
Iwamuro M, Curr Issues Mol Biol 2023 [16]	*Helicobacter pylori* may play a role in the induction/exacerbation of AAG.
Miceli E, Am J Gastroenterol 2023 [20]	The annual rate of progression of 10.9% from “potential” to “overt” AAG would suggest that anti-parietal cell antibodies are a true marker of “potential” AAG in patients without corpus atrophy.
Guo X, J Clin Med 2023 [21]	Anti-parietal cell antibodies, serum gastrin, PGI/PGII ratio, and vitamin B12 can be useful to identify patients with AAG among those with precancerous conditions, i.e., atrophic gastritis and intestinal metaplasia.
Singh S, Cureus 2023 [22]	Patients with AAG are often asymptomatic, but they can refer dyspeptic or reflux symptoms.
Conti L, Microorganisms 2020 [23]	Hypochloridria results in alteration of composition of gastric microbiota and patients with AAG have higher microbial diversity.
Miceli E, Dig Liv Dis 2023 [24]	Atrophic autoimmune gastritis can be linked to infertility, recurrent miscarriage, congenital abnormalities, and several obstetric complications.
Rugge M, GUT 2023 [25]	In patients with AAG, the cumulative incidence of type 1 neuroendocrine tumors (NETs) is 4.7% at 2 years of follow-up.
Dilaghi E, Am J Gastroenterol 2023 [26]	The incidence rate of gastric cancer/high grade dysplasia is 0.5% per person/year; risk factors for gastric cancer are age >60 years, intestinal metaplasia with absence of pseudopyloric metaplasia, pernicious anemia.

AAG: atrophic autoimmune gastritis.
